# The Effect of COVID-19 Safety Protocols on Hospital Workers’ Mental Health: A Moderated-Mediation Model of COVID-19 Anxiety and Psychological Resilience

**DOI:** 10.3390/bs12120477

**Published:** 2022-11-25

**Authors:** Fang Yin, Zhanchun Feng

**Affiliations:** School of Medicine and Health Management, Tongji Medical College, Huazhong University of Science and Technology, Wuhan 430030, China

**Keywords:** mental health, stigma, psychological resilience, COVID-19 safety protocols, COVID-19 anxiety, hospital workers

## Abstract

Social distancing measures can create psychological issues, especially among hospital staff who constantly deal with emergency patients. To explore the mediating role of COVID-19 anxiety on the association between COVID-19 safety protocols and mental health, and to test the moderating role of resilience between COVID-19 safety protocols and COVID-19 anxiety, this work collected data on hospital staff in terms of COVID-19 safety protocols, psychological resilience, COVID-19 anxiety, and improving staff mental health. The effects of the use of COVID-19 safety protocols on COVID-19 anxiety and the mental health of hospital workers in China were also analyzed. The experimental results showed that resilience remarkably moderated COVID-19 safety protocols and COVID-19 anxiety among Chinese hospital staff.

## 1. Introduction

Healthcare workers worldwide face the mental health stigma that substantiates the false notion that “physicians are superhuman beings”. In reality, they also suffer from mental health issues. In the recent decade, the world has not faced an event worse than the COVID-19 pandemic. No one imagined this disease would affect all human activities in such an unprecedented and chaotic manner. This pandemic posed severe challenges to the world population’s social, economic, and psychological resources. One of the defense lines between COVID-19 and society were healthcare workers [1,2,3]; however, their health [4] and social lives were particularly troubled [5]. Interminably, the COVID outbreak attained the status of pandemic [6]. Health workers and administrative authorities worldwide made persistent efforts to alleviate the continuous spread of the COVID-19 virus [7].

The coping strategies to alleviate, or stay safe from, the virus included regularly washing hands and face, keeping a distance from other people, especially at social gatherings, and wearing a mask [8]. Unhealthy coping strategies were also employed by many people, such as smoking, eating more fast food, and taking things injurious to health [9]. These latter coping mechanisms could result in sleeplessness, unhappiness, or other mental health-related conditions [10]. Auxiliary literature has identified a relationship between the COVID-19 pandemic and mental health [4].

To prevent the COVID-19 pandemic, the WHO, and health specialists, advised specific safety protocols, such as hand washing, maintaining distance from other people, wearing a face mask, and quarantining [11,12].

Any country’s primary preference is to provide its people with basic standards of living, which include education, food, justice, and health safety. The prosperity of any country can be assessed from these factors. Efficient healthcare facilities are one of the most important factors [13]. The performance of healthcare workers is based on factors such as financial rewards, organizational support, and mental peace [14]. COVID-19 drastically affected people’s mobility and it spread rapidly with no cure. It caused severe mental health issues, particularly in healthcare workers who worked day and night to attend to patients in hospitals.

According to the National Health Commission of the People’s Republic of China, 3387 out of 77,262 patients with COVID-19 (4.4%) in China were healthcare professionals or worked in medical institutions [15]. According to the Chinese Red Cross Foundation, the People’s Republic of China’s National Health Commission, and public media, 3387 people infected with COVID-19 died due to this infection while practicing medicine in Wuhan and elsewhere in China [16,17,18]. On the basis of these facts, healthcare workers were at a high risk of acquiring COVID-19. Owing to the rising COVID-19 issues and related diseases, the number of healthcare facilities and hospitals in China increased to cope with the situation.

To resolve and overcome the situation, China established several hospitals, designed protocols, and safety measures, and applied some potential strategies to prevent the pandemic [19,20,21]. This study highlights the importance of the mental health concerns of healthcare workers. It helps hospital administrations and hospitals per se revamp their policies by focusing on the factors related to safety measures and psychological resilience concerns so as to overcome mental health issues and reduce employee anxiety.

Stigmatizing the mental health of healthcare workers is a problem that must be addressed. Global healthcare administrations have implemented special response strategies to contain the spread of the COVID-19 pandemic. However, social distancing measures can cause psychological issues, especially among hospital staff who constantly deal with emergency patients. The mental health of hospital staff is an important factor in surviving the challenging situation during COVID-19, and this aspect requires the attention of researchers. There are many studies on the effects of COVID-19 on mental health, but there is a lack of research on the relationships between COVID-19 safety protocols, COVID-19 anxiety, mental health and psychological resilience in hospital workers, and there is insufficient potential evidence on the mediating effects of COVID-19 anxiety and the moderating effects of psychological resilience. Based on this, this study constructed a structural equation model to comprehensively analyze the degree of consistency of surveyed data relationships to form a model of the relationship between COVID-19 safety protocols, COVID-19 anxiety, mental health and psychological resilience. Furthermore, this article examined the mediating effects of COVID-19 anxiety between COVID-19 safety protocols and mental health, and the moderating effects of psychological resilience between COVID-19 safety protocols and COVID-19 anxiety, aiming to provide important evidence for the development of resilience-based mental health interventions for hospital workers. This paper is further divided into multiple sections. Section 2 provides evidence regarding COVID-19 safety protocol, COVID-19 anxiety, mental health, and psychological resilience from previous studies, and elaborates the methodology applied to collect the data and analyze the validity of the data. Section 3 describes the empirical analysis conducted. Section 4 interprets the analysis results. Finally, the last section contains the summary of the study’s results and an outlook for future research.

## 2. Materials and Methods

### 2.1. Hypothesis Development

Technological advancement in the medical field provided safety measures and cures for prevalent diseases. Diseases such as COVID-19 emerge and severely impact the health of people and workers. Any disease or pandemic greatly impacts people’s lives and economic conditions [22,23]. Kaye et al. enumerated the elective procedures of safety protocols and measures that were efficacious in reducing the dominance of COVID-19 anxiety among hospital workers [24,25,26]. The pandemic impacted the global financial situation, by raising the inflation level of almost all commodities. The increase in anxiety levels during COVID-19 disrupted everyday lives of people. The WHO positively introduced safety protocols comprising the use of various aspects of dressings, masks, distancing, and precautionary medication for health and hospital workers.

Yim et al. analyzed the strategies of maintaining safety measures and prerequisites in the hospitals during the COVID-19 pandemic to eliminate anxiety [27]. The COVID-19 safety protocols were found to have a positive effect on people, specifically hospital workers, because they could save many lives. These safety protocols were initially organized when COVID-19 emerged in China. However, the anxiety levels were enhanced due to the closure of businesses and the need to maintain distance. This enhancement negatively influenced hospital workers’ mental health, because they could not meet with their loved ones. Even though the hospital workers were restrained, a ban was imposed on movements from the hospitals. Therefore, the hospital workers faced tremendous anxiety and depression that disrupted their everyday lives and their relationships with their family members.

Siegel et al. examined the symptoms of stress, emotions, sensitivity, and anxiety during COVID-19 to determine the mental health problems [28]. COVID-19 anxiety increased among hospital workers, and many of their family members suffered from depression and anxiety. The pandemic negatively impacted the life and health of hospital workers, and many suffered from other diseases. Safety protocols had a positive role in reducing anxiety, but the fear of disease was too prominent to eliminate all the gaps that could reduce the anxiety levels. Thus, the hypothesis derived from the above discussion is as follows:

**H1:**  *COVID-19 safety protocols significantly influence COVID-19 anxiety.*

Every individual in the world experiences anxiety at some point, whether it pertains to life or professionalism [29]. Anxiety is a feeling individuals encounter in performing tasks in their lives. Improper availability of time also leads to anxiety. Some people have anxiety due to diseases or the diseases of their loved ones. Mohanty et al. investigated the attitudes associated with COVID-19 anxiety that intentionally impacted the mental health of hospital workers where precautionary measures were not adopted [30]. Mattila et al. enumerated the associated factors of COVID-19 anxiety that dominantly prevailed among hospital workers and influenced their mental health [31]. The diseases were less infectious than the fear. These immediate effects were compromised by some international news that sudden death due to COVID-19 was unstoppable and a patient would not have time after receiving a positive result.

The predictors of mental health help in understanding the reasons for psychological anxiety among health workers. Caycho-Rodríguez et al. discussed the predictors of mental health that primarily affected hospital workers and increased the levels of COVID-19 anxiety [32]. Most types of COVID-19 anxiety are unpredictable. Some people experience anxiety due to their illness, and others experience anxiety as a result of the illness of their loved ones. Munyenyembe and Chen assessed quantitative COVID-19 anxiety strategies among health workers in controlling mental health [33]. These negative impacts were the primary cause of COVID-19 anxiety that subsequently increased and disturbed hospital workers’ mental health.

The emotions of hospital workers were deadened during the first wave of COVID-19 in China, and people’s experiences were too punitive. Mittal et al. reported that during the waves of COVID-19, humanity faced a massive loss of mental strength that essentially indicated its future impact on hospital staff and workers [34]. The hospital staff felt increasing levels of anxiety concerning patients, and many patients also died due to the same anxiety. This phenomenon diverted attention to other hospital workers who adopted preventive measures. Thus, the hypothesis derived from the above discussion is as follows:

**H2:**  *COVID-19 anxiety significantly influences mental health.*

Anxiety is a dangerous element of human life that may cause additional damage to the organs of the human body. When people feel anxious for specific reasons, some body organs stop working, leading to dangerous health situations. Different aspects, such as exercise and artificial working, have highlighted the negativity of anxiety and unveiled the factors that could remove anxiety. Mohlman et al. explored COVID-19 inventories that were important in measuring the anxieties linked with the illness and mental health of hospital staff and workers [35]. The human mind entertains hopes and situations which, when positively interpreted, can eliminate anxiety. COVID-19 is a dangerous disease that occurred in 2019 and impacted the whole world with depression and anxiety [36]. Hagan et al. analyzed the mandate of vaccines as a safety measure and protocol during COVID-19 among hospital workers in a social justice policy [37]. Anxiety affected the mental health and safety protocols, and clarifying it could safely manage the implications of safety protocols and stabilize mental health.

COVID-19 anxiety is one of the core elements that resulted in imbalance in the work–life balance of healthcare workers, and caused a lack of focus on professional obligations. It also affected the relationship between mental health of hospital workers and COVID-19 safety protocols. Karatepe et al. narrated the relationship between COVID-19 anxiety, stress, and mental health problems that could be addressed with proper safety protocols [38]. People with solid psychological foundations controlled their anxiety levels by adapting safety protocols. Meanwhile, people with less solid psychological abilities could not control their anxiety by mediating between safety protocols and mental health.

Masjoudi et al. investigated the relationship among the elements COVID-19 anxiety, fear, and stress that favored self-care and helped in recovering from mental health disturbance [39]. Muscular disorders prevailed due to the massive levels of anxiety. Uzunova et al. assessed the management and presentation of COVID-19 anxiety among hospital workers and individuals that significantly helped them recover from adverse mental health [40]. Panic attacks were on the rise due to the obsessional behaviors of patients and hospital workers in response to COVID-19. These behaviors played a negative role in increasing the levels of COVID-19 anxiety that disrupted the mental health of many hospital workers. Good and controlled COVID-19 anxiety could improve the application of safety protocols in a mediating way to save mental health. Thus, the hypothesis derived from the above discussion is as follows:

**H3:**  *COVID-19 anxiety significantly mediates the relationship between COVID-19 safety protocols and mental health.*

Safety protocols and anxiety during COVID-19 are interlinked with each other. Safety protocols play a vital role in the removal of COVID-19 anxiety. These safety protocols were established during the start of COVID-19 to restrain the effects of COVID-19 on unaffected people. Sakız and Aftab investigated the importance of psychological resilience and its dominant characteristics in adopting COVID-19 safety protocols for to control COVID-19 anxiety [41]. The COVID-19 safety protocols provided feasible measures for people and hospital workers. These feasible safety measures involved various curative medications and items of clothing that helped hospital workers protect themselves from the infection.

Sharpley et al. asserted the effects of psychological resilience on the interactions between COVID-19 safety protocols and mental health counseling in hospital workers [42]. Psychological resilience is crucial in COVID-19 anxiety and COVID-19 safety protocols. People with psychological resilience are eminently able in dealing with elements of anxiety. Hospital workers exhibiting psychological springiness are better able to deal with diseases. Carrnar rated the elements of protective glasses, masks, and social distancing COVID-19 safety protocols as important in decreasing anxiety through psychological resilience [43].

Psychological resilience is essential regarding COVID-19 due to the impact of fear of it on health. Psychological resilience in hospital workers gave them the capability and ability to behave effectively and the mental process for speedy recovery and promotion of personal abilities [44]. Arslan et al. discussed the effects of psychological resilience on the relationship between anxiety elements and the implications of safety protocols [45] and found its moderating impact on COVID-19 safety protocols and COVID-19 anxiety.

Brown et al. indicated psychological resilience was essential in performing safety protocols to combat COVID-19 anxiety [46]. All the harmful elements of this type of anxiety are effectively managed by inserting safety protocols moderated by psychological resilience. Managing the emotions and impulses of hospital workers is important because of their vital role in eliminating anxiety among patients. Effective management can be achieved by adopting safety protocols to appease COVID-19 anxiety, moderated by psychological resilience. Thus, the hypothesis derived from the above discussion is as follows:

**H4:**  *Psychological resilience significantly moderates between COVID-19 safety protocols and COVID-19 anxiety.*

### 2.2. Data and Empirical Method

The article examined the impact of COVID-19 safety protocols on COVID-19 anxiety and its impact on the mental health of hospital workers. It also analyzed the moderating impact of psychological resilience regarding COVID-19 safety protocols and COVID-19 anxiety among hospital/health workers in China.

The employees of the designated treatment institutions for patients with COVID-19 in Chongqing China were the respondents.

There were two ways to distribute the questionnaire. First, the researchers directly distributed the questionnaire to the designated medical institutions for COVID-19. Second, the researchers mailed the questionnaire to worker at the designated medical institutions, who assisted the researchers in organizing colleagues to complete the questionnaire. After filling in the questionnaire, the respondent returned it to the author. Both methods were used. Before the questionnaire was issued, the subjects were informed of the important impact of the questionnaire on the research results, and the subjects were required to fill in the questionnaire carefully. At the same time, a small gift was given to each subject to arouse their attention. The author answered any questions concerning the questionnaire as soon as possible. Due to medical workers being extremely busy, the researchers sent 645 questionnaires and received 370 responses, representing around a 57.36 percent response rate. The specifics of the questionnaire are provided in Appendix A.

The questionnaire consisted of the following four parts: basic information of the respondents, views on COVID-19 Safety Protocols, COVID-19 Anxiety status, and Mental Health status and Psychological Resilience. The demographic characteristics of the respondents included sex, age, education, professional title and years of service. The demographic information of the respondents is given in Table 1.

The other three parts of the questionnaires had a five-point Likert scale from one for strongly disagree to five for strongly agree. The items were adopted from past studies. COVID-19 safety protocols had eight items, extracted from Taylor et al. [47], COVID-19 anxiety had five items, taken from Lee [48], mental health also had five items, taken from Berwick et al. [49], and psychological resilience had twelve items, extracted from Scrivner et al. [50]. The measurements and sources are given in Table 2.

To test the pre-specified hypothesis, SEM was used for analysis, and the statistical software AMOS was used. The article applied AMOS to test the hypotheses and associations among variables. AMOS is an effective statistical tool for dealing with large and small sample sizes. In addition, it is also an effective tool that deals with complex models. It checks the reliability of the items and variables using measurement model assessment. The items’ reliability is checked using convergent validity tests like factor loadings, Alpha, and average variance extracted (AVE). The factor loadings and AVE values should be more than 0.50, while Alpha values should be more than 0.70. In addition, variables’ reliability was examined using a discriminant validity test like Fornell–Larcker, and the criteria were that the first value of the column should be larger than the other values in the same column [51].

**Figure 1:**
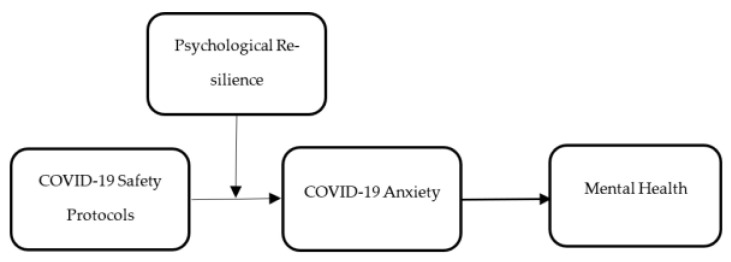
Theoretical framework.

Moreover, good model fitness was also checked using the Tucker–Lewis index (TLI). The criteria for this test were that the value should be higher than 0.90. The model was also checked with the comparative fit index (CFI), and the criteria for the test was that the value should be higher than 0.90. Root mean square error of approximation (RMSEA) was applied and the criteria for the test was that the value should not be higher than 0.10 [52]. Finally, the association among the variables was been examined using structural model assessment, and t-values and *p*-values exposed the significance of the relationships. The relationship was significant if the t-values were more prominent than 1.64 and *p*-values were less than 0.05. Finally, the current study used the COVID-19 safety protocols (CSPs) as the independent variable, COVID-19 anxiety (CAN) as the mediating variable, mental health (MH) as the dependent variable, and psychological resilience (PYR) as the moderating variable. These constructs are given in Figure 1.

## 3. Results

The results show the convergent validity examination using factor loadings, AVE and Alpha. The standard criteria for factor loadings and AVE should be more than 0.50, while Alpha should be more than 0.70. The results indicated that the factor loading values were larger than 0.50, Alpha values were larger than 0.70, Ave values were more than 0.50, and Maximum Shared Variance (MSV) was lower than AVE. The values proved that the convergent validity was valid. Table 3 shows the results.

In addition, the variables’ reliability was examined using a discriminant validity test, Fornell–Larcker, and the criteria were that the first value of the column should be larger than the other values in the same column. The results revealed that the column’s first value was larger than the other values in the same column, and these values exposed the association with the variable as more substantial than with the other variables and provided valid discriminant validity. Table 4 shows these findings.

**Figure 2:**
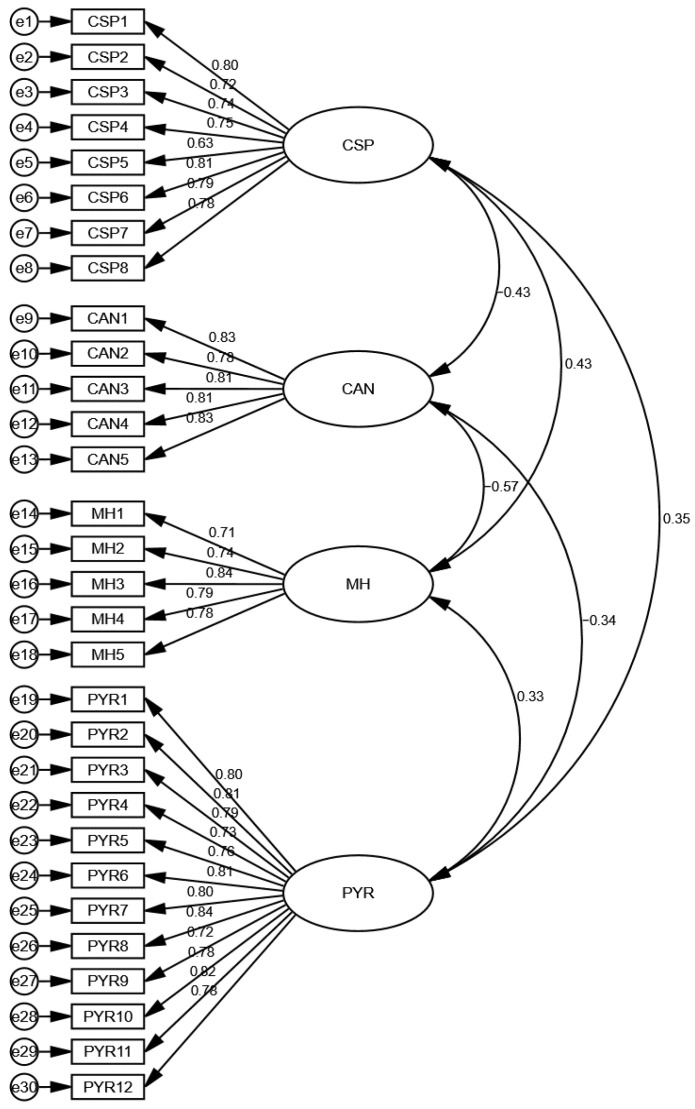
Measurement model assessment.

The results also showed the model’s good fitness using TLI, and the criteria for this test was that the value should be higher than 0.90, and the results showed this to be the case. In addition, the model’s good fitness was checked with CFI, and the criteria for the test was that the value should be higher than 0.90, and the results showed this to be the case. Finally, the model’s good fitness was checked with RMSEA, and the criteria for the test was that the value should not be higher than 0.10, and the results showed this to be the case. Thus, the results exposed that the model was a good fit. These figures are shown in Table 5 and Figure 2.

The results revealed that the COVID-19 safety protocols were negatively linked with COVID-19 anxiety and so H1 was accepted as true. In addition, the results also revealed that COVID-19 anxiety had a negative relationship with the mental health of the hospital workers in China and so H2 was accepted. The findings also indicated that psychological resilience significantly moderates COVID-19 safety protocols and COVID-19 anxiety among hospital workers in China and so H4 was accepted. These relationships are shown in Table 6.

The bootstrapping method was used to test the mediating effect. Setting a random sample size of 5000, with a confidence interval of 95%, the Bias-Corrected estimation method was applied, and Amos was calculated to obtain the effect values, standard error, and upper and lower confidence intervals. If the confidence intervals did not contain 0, the mediating effect was significant. If the results contained 0, the effect was not significant. From the test results: the 95% upper and lower confidence intervals of the total effect of “CSP→MH” was [0.286, 0.497], which did not contain 0, indicating that the total effect between COVID-19 Safety Protocols and Mental Health was significant with an effect value of 0.397. The 95% upper and lower intervals of the direct effect path “CSP→MH” was [0.106, 0.343], which did not contain 0, indicating that the direct effect between COVID-19 Safety Protocols and Mental Health was significant, with an effect value of 0.230. The 95% upper and lower intervals of the mediated path “CSP→CAN→MH” was [0.107, 0.243], which did not contain 0, indicating a significant mediating effect of COVID-19 Anxiety between COVID-19 Safety Protocols and Mental Health, with an effect value of 0.167. These relationships are shown in Table 7.

The results also showed the indirect effects exposed by the mediation analysis. The results indicated the total, direct and indirect effects of the COVID-19 safety protocols, COVID-19 anxiety, and mental health of the hospital workers in China and H3 was accepted.

From the table of high and low groupings of regulation effects, it can be seen that the value of the effect of CSP on CAN was −0.484 at low PYR and −0.214 at high PYR, so the negative effect of COVID-19 Safety Protocols on COVID-19 Anxiety diminished as Psychological Resilience increased. These relationships are shown in Table 8 and Table 9.

**Figure 3:**
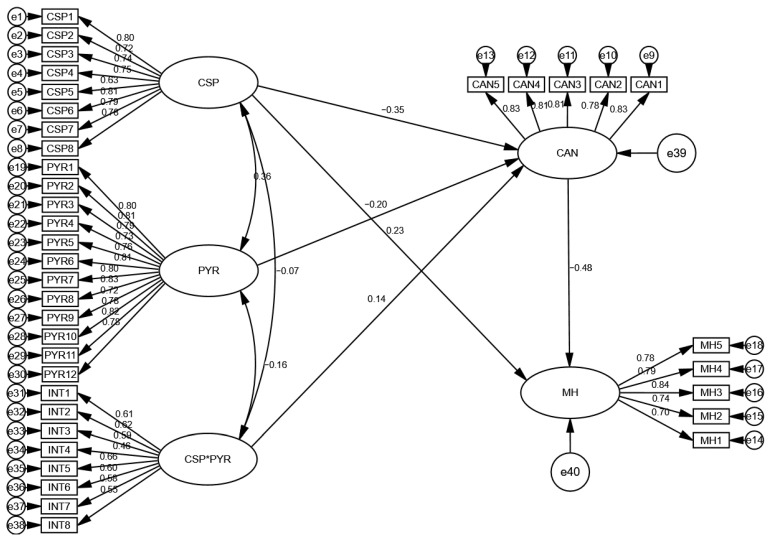
Structural equation model.

Figure 3 shows the path coefficient graph for each path of the model that held significantly. The * in the figure represents the interaction terms.

## 4. Discussion

The study results showed that COVID-19 safety protocols for hospital workers negatively affected COVID-19 anxiety among these workers. In the spread of the COVID-19 pandemic, workers in hospitals or places especially declared as providing medical services had to face the fear of getting infected, as they were working among infected persons. This fear caused anxiety and influenced the workers’ efficiency during their work. Providing COVID-19 safety protocols reduced the fear of getting infected, and the resultant anxiety also reduced. These results were supported by Magnavita et al. [53], who examined the safety protocols for workers in health centers during the COVID-19 pandemic. The study implied that when safety protocols, like gloves, surgical masks, eye protectors, face shields, shoe and head covers, and gowns, were provided to caregivers, the anxiety aroused from the fear of getting infected was released. These results were also supported by Givi et al. [54], who highlighted that COVID-19 was fast becoming a fatal disease which could be spread through the touching of infected persons and infected things, breathing in infected air, or getting drops of fluid from an infected person’s mouth, nose, or other body organs. In such a situation, workers in hospitals who provided care services to patients suffering from COVID-19 were anxious of becoming the victim of the disease during the care process.

The results indicated that COVID-19 anxiety in hospital workers negatively affected workers’ mental health. This anxiety created restlessness in the worker’s minds, destroyed their sense of peace of mind, and affected cognitive capabilities. These results were supported by Sabetian et al. [55], who highlighted that hospital workers assigned the duty to help cure COVID-19 patients and those giving care services had to be mentally healthy so that they could pay attention to their duties and actively perform them. However, many who felt the danger of health infection were anxious about getting infected by the virus. The increase in COVID-19 anxiety disturbed the thinking of workers who provided healthcare services and destroyed their mental health, which affected their performance. Hence, COVID-19 anxiety negatively impacted hospital workers’ mental health. These results were also in line with Liu et al. [56], who argued that workers who had COVID-19 anxiety became the victim of disturbed thinking and suffered from weak mental health.

The results revealed that COVID-19 anxiety is a perfect mediator between COVID-19 safety protocols for hospital workers and their mental health. These results were supported by Cinar et al. [57]. Providing COVID-19 safety protocols reduced this fear of getting infected, and the resultant anxiety reduced. Thus, reducing COVID-19 anxiety built a link between COVID-19 safety protocols for hospital workers and their mental health. These results were also supported by Prakash et al. [58], who showed that when the COVID-19 pandemic broke out many people fell into its cruel clutches, including. the exceptional staff appointed to care for COVID-19 patients in hospitals and healthcare centers. When staff members were provided with COVID-19 safety protocols, they did not succumb to fear and anxiety of also becoming infected. In this situation, the workers enjoyed a high level of satisfaction and, thus, had high mental capacity. These results were also supported by Huang et al. [59], who revealed that COVID-19 safety protocols for hospital workers, COVID-19 anxiety, and mental health of workers were interlinked. COVID-19 safety protocols, like gloves, surgical masks, eye protectors, face shields, shoe and head covers, gowns, and sanitizers, made available to hospital workers reduced COVID-19 anxiety in hospital workers and, thereby, improved their mental health.

The results revealed that psychological resilience moderated the relationship between COVID-19 safety protocols for hospital workers and their COVID-19 anxiety. These results were supported by Hagan et al. [38], who discussed COVID-19 concerns and hospital workers’ performance. The study implied that it was the thinking of individuals in society that affected their actions in the outbreak of the COVID-19 pandemic. Usually, people are not ready to take precautions and are anxious about disease and its consequences. In hospitals, hospital workers had immediate psychological resilience benefit from COVID-19 safety protocols to overcome COVID-19 anxiety.

Hence, psychological resilience moderated the association between COVID-19 safety protocols for hospital workers and reduced COVID-19 anxiety among them. These results were also in line with Ha [60]. This academic article posited that in the case of top management personnel and hospital workers fast psychological resilience was fostered in the provision, and implementation, of COVID-19 safety protocols. Psychological resilience was also helpful in controlling COVID-19 anxiety and giving hope that everything would be fine. Thus, psychological resilience strengthened the relationship between COVID-19 safety protocols for hospital workers and reduction of COVID-19 anxiety.

The study has excellent theoretical significance and contributes to the literature on the COVID-19 pandemic. The study’s primary concern was assessment of the mental health of healthcare workers during COVID-19. The study focused on the role of the COVID-19 safety protocols in improving the mental health of hospital workers. In previous studies, researchers have mainly focused on the role of COVID-19 safety protocols in hospital workers’ physical health, and little attention has been given to mental health. So, the present study analyzed the role of COVID-19 safety protocols in improving the mental health of hospital workers, and is an excellent contribution to the literature. The use of COVID-19 anxiety as a mediator between the role of COVID-19 safety protocols and hospital workers’ mental health, and the role of psychological resilience as a moderator between COVID-19 safety protocols and hospitals’ workers COVID-19 anxiety is also a significant contribution to literature. This study has much more significance in the practical field, as it addresses COVID-19, its impacts, and ways to overcome the negative consequences of COVID-19, which is a worldwide issue. This study is about the mental health of healthcare workers during COVID-19. In a critical situation, when COVID-19, a contagious and potentially fatal disease, was at its peak, the healthcare department and the work efficiency of workers offering their services in this department played a key role in controlling the virus, curing infected persons, and maintaining health score among the public.

## 5. Conclusions

This study demonstrated the mediating effect of COVID-19 anxiety on the association between COVID-19 safety protocols and mental health and tested the moderating role of psychological resilience between COVID-19 safety protocols and COVID-19 anxiety. An empirical research survey was conducted in China to collect data on COVID-19 safety protocols for hospital workers, psychological resilience, COVID-19 anxiety, and improving workers’ mental health. It was also observed that COVID-19 safety protocols directly influenced and reduced anxiety among healthcare workers, leading to better mental health. This, therefore, suggests that COVID-19 safety and protocols have a significant role to play. Additionally, psychologically resilient health workers can deal better with anxiety issues and maintain their mental health. The results also revealed that when COVID-19 spread and caregivers in hospitals had to work among infected people, COVID-19 safety protocols reduced COVID-19 anxiety, and the mental health of hospital workers improved. The study’s results also revealed that improvement in psychological resilience helped in implementing the policy for COVID-19 safety protocols for hospital workers and reduced COVID-19 anxiety. Health experts and communicators may capitalize on these findings to educate people on COVID-19. Future studies are needed to ideally ascertain the extent of safety measures and protocols needed to stimulate, or initiate, better preventive behaviors and healthcare practices.

## Figures and Tables

**Table 1:** Demographic information of the respondents.

Demographic Variables	Category	Population	Percentage
Gender	Male	223	60.27%
	Female	147	39.73%
Age	22–29	53	14.32%
	30–39	157	42.43%
	40–49	139	37.57%
	50–59	21	5.68%
Highest Education	College degree	13	3.51%
	Bachelor degree	137	37.03%
	Master degree	196	52.97%
	Doctor degree	24	6.49%
Professional and Technical Title	Junior Professional Title	56	15.14%
	Intermediate Certificate	222	60.00%
	Associate Senior Title	79	21.35%
	Senior Title	13	3.51%
Years of work	Less than 3 years	33	8.92%
	3 to 5 years	112	30.27%
	5 to 10 years	128	34.59%
	10 to 20 years	67	18.11%
	More than 20 years	30	8.11%

**Table 2:** Measurements of the variables.

Items	Measurements	Sources
**COVID-19 Safety Protocols**		
CSP1	“Healthcare workers who work in hospitals are likely to have COVID-19”.	[47]
CSP2	“For the safety of the community, healthcare workers should not go out in public”.	
CSP3	“Healthcare workers should have some restrictions on their freedom”.	
CSP4	“Healthcare workers who treat people with COVID-19 should be isolated”.	
CSP5	“I do not want to be around healthcare workers who treat COVID-19 patients”.	
CSP6	“I do not want to be around someone who works in a healthcare setting”.	
CSP7	“Healthcare workers who treat people with COVID-19 should be separated from their families”.	
CSP8	“I would not be comfortable visiting a healthcare worker for medical reasons because I would be worried I might get COVID-19”.	
**COVID-19 Anxiety**		
CAN1	“I felt dizzy, lightheaded, or faint when I read or listened to news about the coronavirus”.	[48]
CAN2	“I had trouble falling or staying asleep because I was thinking about the coronavirus”.	
CAN3	“I felt paralyzed or frozen when I thought about, or was exposed to, information about the coronavirus”.	
CAN4	“I lost interest in eating when I thought about, or was exposed to, information about the coronavirus”.	
CAN5	“I felt nauseous or had stomach problems when I thought about, or was exposed to, information about the coronavirus”.	
**Mental Health**		
MH1	“To what extent have you been a very nervous person”.	[49]
MH2	“To what extent have you felt calm and peaceful”.	
MH3	“To what extent have you felt downhearted and blue”.	
MH4	“To what extent have you been a happy person”.	
MH5	“To what extent have you felt so down in the dumps that nothing could cheer you up”.	
**Psychological Resilience**		
PYR1	“During the pandemic, I have been more depressed than usual”.	[50]
PYR2	“Compared to how I usually feel, I have been more nervous and anxious during the pandemic”.	
PYR3	“I am more irritable than usual”.	
PYR4	“I have not been sleeping well since the pandemic started”.	
PYR5	“I have been taking the news about the pandemic in my stride”.	
PYR6	“I have been able to find things to enjoy during the pandemic”.	
PYR7	“I feel positive about the future”.	
PYR8	“I have found some aspects of the pandemic to be interesting”.	
PYR9	“I believe in my ability to get through these difficult times”.	
PYR10	“I know that I can get through these uncertain times”.	
PYR11	“Life has felt meaningful during the pandemic”.	
PYR12	“I know I can get through the problems due to the pandemic”.	

**Table 3:** Convergent validity.

Constructs	Items	Loadings	CR	AVE	MSV
COVID-19 Safety Protocols	CSP1	0.801	0.913	0.569	0.188
	CSP2	0.720			
	CSP3	0.736			
	CSP4	0.750			
	CSP5	0.629			
	CSP6	0.810			
	CSP7	0.792			
	CSP8	0.781			
COVID-19 Anxiety	CAN1	0.834	0.907	0.662	0.330
	CAN2	0.780			
	CAN3	0.813			
	CAN4	0.810			
	CAN5	0.831			
Mental Health	MH1	0.707	0.881	0.598	0.330
	MH2	0.736			
	MH3	0.844			
	MH4	0.793			
	MH5	0.781			
Psychological Resilience	PYR2	0.809	0.951	0.619	0.125
	PYR3	0.793			
	PYR4	0.733			
	PYR5	0.764			
	PYR6	0.809			
	PYR7	0.797			
	PYR8	0.835			
	PYR9	0.719			
	PYR10	0.776			
	PYR11	0.821			
	PYR1	0.800			
	PYR12	0.782			

**Table 4:** Discriminant validity.

Latent Variables	CSP	CAN	MH	PYR
CSP	0.754			
CAN	−0.431	0.814		
MH	0.434	−0.575	0.773	
PYR	0.354	−0.341	0.334	0.787

**Table 5:** Goodness−of−fit Indices.

Selected Indices	Result	Acceptable Level of Fit
TLI	0.954	TLI > 0.90
CFI	0.957	CFI > 0.90
RMSEA	0.038	RMSEA < 0.05 good; 0.05 to 0.10 acceptable

**Table 6:** Direct Path.

Relationships	Beta	Std. Beta	S.E.	C.R.	*p*
CAN←CSP	−0.375	−0.349	0.062	−6.068	***
CAN←PYR	−0.196	−0.200	0.054	−3.609	***
CAN←Interaction Term	0.176	0.137	0.072	2.452	0.014
MH←CAN	−0.448	−0.479	0.058	−7.706	***
MH←CSP	0.231	0.230	0.056	4.091	***

Note: ← means path of direction; *** means it is less than 0.001.

**Table 7:** Mediation Analysis.

	Effect Value	S.E.	Bootstrapping (N = 5000)	
			95%CI	
Total Effect	0.397	0.054	0.286	0.497
Direct Effect	0.230	0.060	0.106	0.343
Indirect Effect	0.167	0.035	0.107	0.243

**Table 8:** Regulating effect of high and low groupings.

Group	Diresct Effect of CSP→CAN	S.E.	95%CI	
			Lower	Upper
Low PYR (M − SD)	−0.484	0.081	−0.640	−0.319
Mean PYR (M)	−0.349	0.058	−0.459	−0.231
High PYR (M + SD)	−0.214	0.092	−0.390	−0.030

**Table 9:** High and low groupings of mediating effects with regulation.

Group	Indirect Effect of CSP→CAN→MH	S.E.	95%CI	
			Lower	Upper
Low PYR (M − SD)	0.232	0.048	0.148	0.339
Mean PYR (M)	0.167	0.035	0.107	0.243
High PYR (M + SD)	0.102	0.046	0.018	0.200

## Data Availability

The datasets generated and analyzed in this study are available from the corresponding author upon reasonable request.

## Appendix A. Questionnaire on the Effect of COVID-19 Safety Protocols on Hospital Workers’ Mental Health

Dear Doctors,

Hello, and thank you for participating in this survey. The purpose of the survey is to study Safety Protocols on Hospital Workers’ Mental Health and we promise to use the survey for academic purposes only and not to disclose your personal information. Please answer the survey, based on your real situation, and thank you for taking the time to participate.

Formal questionnaire.					
**Basic information on survey respondents**					
Items	Options				
1. Gender	A. Male B. Female				
2. Age	A. 22–29 B. 30–39 C. 40–49 D. 50–59				
3. Highest Education	A. College degree B. Bachelor degree C. Master degree D. Doctor degree				
4. Professional and Technical Title	A. Junior Professional Title B. Intermediate Certificate C. Associate Senior Title D. Senior Title				
5. Years of work	A. Less than 3 years B. 3 to 5 years C. 5 to 10 years D. 10 to 20 years				
**Views on COVID-19 Safety Protocols, COVID-19 Anxiety status, Mental Health status and Psychological Resilience**					
**opic\Options**	**Strongly Agree**	**Somewhat Agree**	**Moderately**	**Somewhat Disagree**	**Disagree**
**COVID-19 Safety Protocols**					
Healthcare workers who work in hospitals are likely to have COVID-19.					
For the safety of the community, healthcare workers should not go out in public.					
Healthcare workers should have some restrictions on their freedom.					
Healthcare workers who treat people with COVID-19 should be isolated.					
I do not want to be around healthcare workers who treat COVID-19 patients.					
I do not want to be around someone who works in a healthcare setting.					
Healthcare workers who treat people with COVID-19 should be separated from their families.					
I would not be comfortable visiting a healthcare worker for medical reasons because I would be worried I might get COVID-19.					
**COVID-19 Anxiety**					
I felt dizzy, lightheaded, or faint when I read or listened to news about the coronavirus.					
I had trouble falling or staying asleep because I was thinking about the coronavirus.					
I felt paralyzed or frozen when I thought about, or was exposed to, information about the coronavirus.					
I lost interest in eating when I thought about, or was exposed to, information about the coronavirus.					
I felt nauseous or had stomach problems when I thought about, or was exposed to, information about the coronavirus.					
**Mental Health**					
To what extent have you been a very nervous person.					
To what extent have you felt calm and peaceful.					
To what extent have you felt downhearted and blue.					
To what extent have you been a happy person.					
To what extent have you felt so down in the dumps that nothing could cheer you up.					
**Psychological Resilience**					
During the pandemic, I have been more depressed than usual.					
Compared to how I usually feel, I have been more nervous and anxious during the pandemic.					
I am more irritable than usual.					
I have not been sleeping well since the pandemic started.					
I have been taking the news about the pandemic in my stride.					
I have been able to find things to enjoy during the pandemic.					
I feel positive about the future.					
I have found some aspects of the pandemic to be interesting.					
I believe in my ability to get through these difficult times.					
I know that I can get through these uncertain times.					
Life has felt meaningful during the pandemic.					
I know I can get through the problems due to the pandemic.					
